# Reverting Immune Suppression to Enhance Cancer Immunotherapy

**DOI:** 10.3389/fonc.2019.01554

**Published:** 2020-01-21

**Authors:** Bella S. Guerrouahen, Cristina Maccalli, Chiara Cugno, Sergio Rutella, Emmanuel T. Akporiaye

**Affiliations:** ^1^Sidra Medicine, Member of Qatar Foundation, Research Department, Doha, Qatar; ^2^John van Geest Cancer Research Centre, Nottingham Trent University, Nottingham, United Kingdom; ^3^Veana Therapeutics, Inc., Portland, OR, United States; ^4^Providence Cancer Center, Portland, OR, United States

**Keywords:** immunotherapy, immunosuppression, tumor escape, soluble factors, tumor microenvironment, immune checkpoint inhibitors, immunosuppressive enzymes

## Abstract

Tumors employ strategies to escape immune control. The principle aim of most cancer immunotherapies is to restore effective immune surveillance. Among the different processes regulating immune escape, tumor microenvironment-associated soluble factors, and/or cell surface-bound molecules are mostly responsible for dysfunctional activity of tumor-specific CD8^+^T cells. These dynamic immunosuppressive networks prevent tumor rejection at several levels, limiting also the success of immunotherapies. Nevertheless, the recent clinical development of immune checkpoint inhibitors or of molecules modulating cellular targets and immunosuppressive enzymes highlights the great potential of approaches based on the selective disruption of immunosuppressive networks. Currently, the administration of different categories of immunotherapy in combination regimens is the ultimate modality for impacting the survival of cancer patients. With the advent of immune checkpoint inhibitors, designed to mount an effective antitumor immune response, profound changes occurred in cancer immunotherapy: from a global stimulation of the immune system to a specific targeting of an immune component. This review will specifically highlight the players, the mechanisms limiting an efficient antitumor response and the current immunotherapy modalities tailored to target immune suppressive pathways. We also discuss the ongoing challenges encountered by these strategies and provide suggestions for circumventing hurdles to new immunotherapeutic approaches, including the use of relevant biomarkers in the optimization of immunotherapy regimens and the identification of patients who can benefit from defined immune-based approaches.

Cancer growth and progression is controlled by the immune cells infiltrating the tumor microenvironment (TME). Several reports provide clear evidence that activation of an antitumor immune response in the host results in tumor regression and translates into better clinical outcomes in animal and human cancers (1, 2). However, more often than not, the interactions between the immunological players and the tumor cells in the TME lead to immune evasion contributing to tumor progression (3). Importantly, the immune selection inadvertently favors the emergence of tumors with reduced immunogenicity. The stromal compartment is required to create a permissive environment for the extravasation and spread of genetically and epigenetically altered tumor cells (4), and maintain the inactivation of various components of the immune system, preventing their adequate functioning (5). The major role of the immune system in oncology has been highlighted by the identification of one of the cancer hallmarks: “tumor evasion from immune surveillance” (4). Instead of using specific inhibitors to target tumor cells, immunotherapy drugs facilitate either the generation of anti-tumor immune responses or the unleashing of the patient's immune system against cancer through immune checkpoint blockade (ICB). Co-inhibitory molecules are crucial players in the regulation of T cell responses by modulating the signaling cascade initiated by T cell receptor (TCR) engagement. An antigen-independent “second signal” is required to counteract T cell activation and to induce a down-modulation of the immune system activation toward a resting state (6). ICB functions as tumor suppressing factors through the modulation of immune cell-tumor cell interaction, preventing an effective tumor attack. Immunotherapy strives to improve immune system functions, and therefore potentiate immune surveillance of cancer resulting in effective tumor control. Unfortunately, the response to treatment and the course of the disease can be influenced by several elements including the capacity of the tumor to adapt through loss of immunogenicity, the impairment of tumor antigen processing, and presentation, and the modulation of the TME toward immunosuppression. The immune contexture defined by the presence of immune-suppressive or -regulatory cell types of the adaptive and innate immune system, or the production of immunosuppressive factors may result in the impairment of local tumor effector cells that can result also in limited systemic anti-tumor immune responses and tumor progression (7). The tumor progression is primarily due to the deleterious effects of tumor- and cell- derived factors and co-inhibitory molecules present in the TME. Understanding and overcoming these tumor escape mechanisms remain a challenge for the successful treatment of cancer. In this review, factors and molecules in the TME, and the strategies that neutralize their effects will be addressed. Results from ongoing immunotherapy clinical studies and agents that have been recently identified as standard care for some type of tumors will be summarized.

## Soluble Factors

### Interleukin-10 (IL-10)

IL-10, identified as cytokine synthesis inhibitory factor (CSIF) is a potent anti-inflammatory cytokine which is structurally related to interferon (IFN)-γ (8). IL-10 is produced by various types of immune cells, including T regulatory cells (Tregs), T helper cells (Th)-1, Th17, B cells, activated monocytes, macrophages, mast cells, granulocytes, dendritic cells (DC) and tumor cells, thus preventing the inflammatory environment created by cancer (9, 10). Recognized as a Th2 cytokine, IL-10 secretion influences the dysfunction of innate and adaptive immunity to allow the escape of malignant cells from immune surveillance by inhibiting the Th1 immune response and the T cell cytotoxic activity (11). IL-10 signaling suppresses T cell function by stimulating the transcription of genes known to suppress toll-like receptor (TLR)-dependent and IFN-γ-dependent signaling in antigen presenting cells (APCs), and thus limiting their function. In already activated DCs, IL-10 has no effect, but in activated monocytes/macrophages IL-10 induces March-I, an ubiquitin ligase that affects antigen presentation by limiting the expression of the major histocompatibility complex (MHC)-II and CD86 (12). In a spontaneously metastatic 4T1 mammary carcinoma mouse model, the increase in myeloid-derived suppressor cells (MDSCs) production of IL-10 decreased the macrophage production of IL-12, and thereby impaired tumor immunity (13). In murine models of inflammatory bowel disease, the frequency of DNA mutations in the colon was 4- to 5-fold greater in IL-10 deficient mice than in IL-10-sufficient mice (14). Treatment of IL-10 deficient mice with a pegylated (PEG) form of recombinant human IL-10 increased the cytotoxic activity of CD8^+^ T cells and controlled tumor growth. Moreover, when cured by PEG IL-10, mice had a long-lasting immunity (15, 16).

A meta-analysis revealed that there is a strong correlation between high levels of circulating IL-10 and poor prognosis of various patients with most types of solid tumors and hematological malignancies (17). Using biopsies from patients with oral squamous cell carcinoma, tumor-associated macrophages (TAMs) expressing CD163^+^CD204^+^ promoted T cell apoptosis and immunosuppression via IL-10 and PD-L1 production, thus predicting an unfavorable prognosis (18). Clinical trials are ongoing for evaluating pegylated recombinant human IL-10 (AM0010, pegilodecakin) in combination with pembrolizumab in metastatic non-small-cell lung carcinoma (NSCLC) (ClinicalTrials.gov Identifier: NCT03382899). After a favorable phase Ib trial in pancreatic cancer, a phase III study is evaluating the safety and efficacy of AM0010 in combination with FOLFOX compared to FOLFOX monotherapy in metastatic pancreatic cancers as a second-line therapy (NCT02923921). Up to date, no IL-10 receptor agonist has received regulatory approval for its use.

### Vascular Endothelial Growth Factor (VEGF)

The VEGF family of growth factors includes the splice variant forms: VEGF-A, VEGF-B, VEGF-C, VEGF-D and placental growth factor (PLGF) (19). VEGF-A, often referred to as VEGF, is the predominant ligand for VEGF receptor 2 (VEGFR2, KDR). VEGF-A exerts a potent proangiogenic effect that stimulates endothelial cell proliferation, migration, and survival in both normal and pathological angiogenesis (19, 20). Both tumor and stromal cells in the TME can produce VEGF (19). VEGF affects lymphocyte-endothelium interactions by altering the adhesion molecule clustering process at the endothelial cells surface, thus controlling lymphocyte trafficking (21). Secondly, VEGF has a systemic immunosuppressive effect. Intratumoral VEGF production limits T cell recruitment into tumors, promote T cell exhaustion and induces accumulation of immune-regulatory cells, such as immature DCs, MDSCs, Tregs, and TAMs (22, 23). Increased VEGF serum levels or tumor expression are associated with a poor prognosis in patients with malignancies including metastatic colorectal cancer (24). Interestingly, DCs matured in the presence of VEGF express less human leukocyte antigen-DR (HLA-DR) and CD86. This expression can be restored by VEGF inhibitors, bevacizumab, and sorafenib. VEGF increases also the expression and activity of indoleamine 2,3- dioxygenase (IDO) in DCs, which has a suppressive effect on antigen (Ag)-specific and mitogen-stimulated lymphocyte proliferation (25). Tumor-produced VEGF-A attracts Nrp1-expressing Tregs and this interaction mediates Tregs infiltration into the tumor (26). In ovarian cancer patients, VEGF enhances expression of PD-1 and other inhibitory checkpoints involved in CD8^+^ T cell exhaustion (27). A preclinical report demonstrated *in vitro* inhibition of the tumor growth with a decrease in the density of vessels in tumor-bearing mice treated with monoclonal antibodies targeting and neutralizing VEGF-A (28).

Based on preclinical evidences, bevacizumab (Avastin, Genentech, Inc.) has been approved in 2004 by the U. S. Food and Drug Administration (FDA) for the first-line treatment of metastatic colorectal cancer (29). Although, several inhibitors of VEGF/VEGFR2 (i.e., bevacizumab, pazopanib, sunitinib, sorafenib) are commonly used in the clinic, they are beneficial only to a subset of patients. The limitations are due to several relapse mechanisms occurring during the anti-angiogenic therapies, including an upregulation of PD-L1 by cytotoxic T lymphocytes (CTL)-secreted IFN-γ (30), and abnormalities in the tumor endothelium (31). Multiple trials are currently investigating combinations of angiogenesis inhibitors and immunotherapies in multiple cancers [(32), NCT02443324], and in patients with advanced diseases (NCT02348008, NCT01633970). Bevacizumab treatment combined with carboplatin and paclitaxel received FDA approval in June 2018 for post-surgery treatment of patients with stage II or IV epithelial ovarian, fallopian tube, or primary peritoneal cancer, followed by single-agent bevacizumab. In metastatic melanoma patients, the combination of bevacizumab and ipilimumab induced changes in tumor vasculature, inflammation status, lymphocyte trafficking, and immune regulation. Analysis of the 46-patient cohort demonstrate a median survival >2 years with significant antitumor activity at the maximum tolerated dose (33). Maintenance nivolumab plus bevacizumab has been tested vs. nivolumab monotherapy and showed improved progression free survival (PFS) results (NCT01454102, CheckMate 012). However, in this comparison the nivolumab monotherapy arm comprise patients with squamous and non-squamous histology, while the nivolumab plus bevacizumab arm included only patients with non-squamous histology (median PFS of 16 weeks in squamous patients and 21.4 weeks in non-squamous patients in the nivolumab monotherapy arm compared to a median PFS of 37.1 weeks in the combination arm). No significant variance in the overall survival (OS) was observed in the two different treatment groups. Another phase III clinical trial, comparing the PFS and the OS of nivolumab combined with ipilimumab vs. the VEGF signaling inhibitor sunitinib in previously untreated advanced renal cell carcinoma (RCC) so far showed so far minimal toxicities and a reduction of the frequency of Tregs [NCT02231749, CheckMate 214, (34)].

### Prostaglandin E2 (PGE2)

The bioactive lipid PGE2, product of the conversion of arachidonic acid by cyclooxygenase 2 (COX-2) is synthesized by various cell types, including cancer, stromal, and infiltrating myeloid cells. In the TME, PGE2 mediates its effects by acting on a group of G protein-coupled receptors (EP1-EP4) (35). The involvement of each receptor in regard to immunosuppression has been studied and revealed that EP1 and EP2 are low-affinity receptors and require significantly higher concentrations of PGE2 for effective signaling. EP3 and EP4 are high affinity receptors (35). Most of the immunomodulatory effects of PGE2 on immune cells are mediated through EP2 and EP4 receptors. EP2 and EP4 are Gαs coupled protein and stimulate adenylyl cyclase to raise the intracellular level of cAMP, and thus protein kinase A (PKA) which activate various types of signaling molecules. However, only EP4, mainly expressed on myeloid cells, T lymphocytes, and tumor cells is known to induce T cell factor–mediated transcriptional activity through phosphatidylinositol 3-kinase (PI3K) as well as PKA (36). EP4, additionally contributes to PGE2-mediated enhancement of tumor survival pathways and suppression of antitumor immune responses. PGE2 induces immunosuppression by inhibiting effector functions of macrophages, neutrophils, CTL, Th1 and natural killer (NK) cell-mediated immunity and by directly downregulating the production of Th1 cytokines. PGE2 also stimulates the development of suppressive types of Tregs, Th17, MDSCs and upregulates Th2-associated cytokines (36). PGE2 has the ability to suppress the production of IL-12 in monocytes and DCs, which is essential to Th1 responses (37). In RCC patients, PGE2 induces arginase I production by MDSCs. Subsequently arginine is depleted and T cell signal transduction and function impaired (38). A positive feedback loop between PGE2 and COX2 leads to redirect the differentiation of DCs toward monocytic MDSCs and locally promote the development of suppressive MDSCs (39). Moreover, in the ascites, MDSC precursors locally accumulate in a CXCR4-dependent manner that requires COX2 activity and autocrine PGE2 production (40). Mice deficient in EP2 significantly develop less tumors compared with tumors in wild-type mice (41). In animal models, E7046, a selective small-molecule inhibitor of EP4 showed an immune dependent growth inhibitory activity, and moreover showed synergistic antitumor activity when combined with anti-CTLA-4 antibodies (42).

The recently completed phase I study NCT02540291 assessed the potential of E7046 in patients with cancers that harbor high levels of myeloid infiltrate cells in the TME. The results showed that although the maximum tolerated dose was not reached, E7046 demonstrated an antitumor activity, and immune modulation in tumors and peripheral blood. Another EP4 antagonist, AAT 008, more potent in pharmacokinetic and pharmacodynamic modeling than Grapiprant (AT-001, AAT-007, CJ-023,423) has not yet been tested in clinic (43).

### Colony-Stimulating Factor (CSF-1)

The proinflammatory cytokine CSF-1, also known as macrophage-CSF (M-CSF) binds to the CSF-1 receptor (CSF1R), which belongs to the Type III protein tyrosine kinase receptors. The axis CSF-1/CSF1R is crucial for the differentiation and survival of the mononuclear phagocyte system, and in particular macrophages. Its expression correlates with diminished survival in some cancers, including breast cancer (44, 45). Moreover, elevated CSF-1 levels in the serum of early breast cancer patients predicts poor survival (46). Macrophages are recruited into tumors following activation of CSF1R by either CSF1 or IL-34, two high-affinity ligands. Interestingly, in a cohort of lung cancer patients, the co-expression of CSF-1 with IL-34 in primary tissues correlates with advanced disease stages and poor survival (47). In addition to TAMs, CSF1R is expressed on DCs, neutrophils, and MDSCs. In murine models of cervical and breast carcinomas, inhibition of CSF1R by a highly selective small molecule inhibitor attenuates the turnover rate of TAMs while increasing the number of CD8^+^T cells that infiltrate the tumors (48). Other preclinical studies suggest that targeting of CSF1R pathway in combination with standard of care treatment may strategically be more effective for activating antitumor responses (49).

Inhibition of CSF-1/CSF-1R pathways suppresses the recruitment of circulating monocytes at the tumor site, and the blocking of TAMs activation, as the major survival factors for these macrophages. Small molecules or clinical antibodies against CSF-1, and CSF-1R belong to a new class of immune-modulatory drugs. Several clinical trials evaluating those inhibitors (PLX3397, RG7155, Durvalumab) alone or in combination with immunotherapy agents (anti-PD-1, -PD-L1) or chemotherapies (paclitaxel, fluorouracil, gemcitabine, oxaliplatin, irinotecan) have been completed (NCT02452424, NCT01494688, NCT01346358, NCT02718911). Recently, a study was launched to measure PFS for patients with advanced pancreatic cancer with poor prognosis treated with the IgG4 monoclonal antibody (mAb) blocking CSF-1R (cabiralizumab) combined with nivolumab, with or without chemotherapy (NCT03336216).

### Transforming Growth Factor (TGF)-β

TGF-β is the prototype of the superfamily containing over forty members, including TGF-βs, Nodal, Activin, and bone morphogenetic proteins (BMPs) (50). TGF-βs are composed of a group of three members (TGF-β1, -β2, and -β3) normally involved in a variety of biological processes (50). Frequently, TGF-β refers to the isoform TGF-β1, which represents the most widely studied. Produced in large amounts within the TME by every leukocyte lineage, including lymphocytes, macrophages, and DCs, TGF-β1 is crucial for protecting the body from the development of excessive immune responses, therefore maintaining immune homeostasis (51). Active TGF-β binds to dimeric TGF-β type 2 receptor (TβRII), which recruits and activates a second dimeric type 1 receptor (TβRI) through its serine/threonine kinase to form a complex. The activated receptor initiates the signaling pathways and phosphorylates the transcription factors SMAD2 and SMAD3 that subsequently form a complex with SMAD4 and cofactors to translocate into the nucleus and modulate the expression of target genes. In addition, depending on the pleiotropic nature of TGF-β actions, activated TGF-β receptor complexes can also trigger a SMAD-independent pathway, including MAPK, PI3K/AKT, and Rho-like GTPase signaling pathways. (52). In the context of cancer, TGF-β play a pivotal role depending on the stage of the tumor (50). In the phase of pre-malignant cells, the tumor suppressive role of TGF-β interferes with tumor proliferation and progression. TGF-β promotes the immunological tolerance by directly suppressing the cytolytic activity of NK and CTLs, repressing the transcription of genes encoding key proteins (such as perforin, granzymes, and cytotoxins), and controlling the inflammatory responses through the regulation of chemotaxis, activation, and survival of lymphocytes, macrophages, granulocytes, NK cells, DCs and mast cells (53, 54). TGF-β is also instrumental for converting conventional CD4^+^ T cells to “induced” FoxP3^+^ Tregs (55), recruiting and stimulating the expansion of the mediators of *in vivo* suppression, MDSCs and Tregs. Recently, it has also been suggested that tumor immune evasion can occur by TGF-β-driven conversion of NK cells into type 1 innate lymphoid cells by an unknown mechanism (56). However, in late stage of cancer development, elevated levels of TGF-β favors tumor progression via effects on the stroma, induction of angiogenesis and/or promotion of the epithelial-to-mesenchymal transition (57–59). The explanation for these contradictory roles is that some tumors develop TGF-β-inactivating mutations and progress in a TGF-β-independent manner, while other tumors accumulate mutations in tumor suppressor genes downstream of TGF-β signaling. Cancer cells that acquire these mutations gain a great advantage over their non-mutated counterparts, as they can exploit the wide range of pro-tumorigenic effectors downstream of TGF-β stimulation (60). Increased systemic ligand levels of TGF-β and aberrant TGFβ signaling are often correlated with aggressive disease and poor prognosis (61). In preclinical studies, targeting the TGF-β pathway may be achieved through the use of several agents, including antisense oligonucleotides, TGF-β-neutralizing antibodies and TGF-β receptor kinase inhibitors (52). In some models of tumor-bearing mice, blockade of the immunomodulator TGF-β with antibodies or genetic manipulation decrease the number of induced Tregs (62). In mice expressing the four main mutations associated with colorectal cancer, the blockade of TGF signaling sensitized the tumor to the action of anti–PD-L1 antibodies (63). In a model replicating the immune-excluded phenotype, co-administration of anti-PDL1 and anti-TGF-β therapies reduced TGFβ signaling in stromal cells, and thus increased the ability of T cells to infiltrate tumors (64). Although, the microenvironment-targeted strategy targeting TGF-β pathway is being well tolerated in preclinical studies, translation into patients remains challenging and raises concerns, which include the development of autoimmune toxicities, the targeting of other homeostatic functions such as angiogenesis, and the risk of developing new malignancies.

Early phases clinical trials using TGF-β monotherapy have yielded conflicting results. While, one study using a monoclonal blocking antibody specific for TGF-β1 reported no clear evidences of antitumor effects in patients despite no major adverse side effects (65), other evaluations showed encouraging results. An mAb neutralizing all isoforms of TGF-β1, GC1008 (Fresolimumab) demonstrated preliminary evidence of anti-tumor effects in a subgroup of patients with advanced malignant melanoma and RCC (66). Interestingly, metastatic breast cancer patients treated with high dose fresolimumab during radiotherapy had a favorable systemic immune response, and thus, a better prognosis (67). Preliminary data from a phase I dose-escalation study suggested that M7824 (MSB0011359C), a bifunctional fusion protein composed of a chimeric version of an anti-PD-L1 mAb with a fragment of TGF-βR4 to entrap active TGF-β, has demonstrated a favorable safety profile in patients with pre-treated advanced solid tumors [NCT02517398, (68)]. Galunisertib (LY2157299), a small molecule antagonist of TGFβ-R1 is being studied in combination with an anti-PD-L1 in NSCLC, hepatocellular carcinoma, pancreatic cancer (NCT02423343, NCT02734160) and with paclitaxel in triple negative breast cancer (NCT02672475).

## Enzymes

### Indolamin 2, 3-Dioxygenase

IDO is an IFN-γ-inducible metabolic enzyme localized in the cell cytoplasm. IDO catalyzes the breakdown of tryptophan (Trp), an essential amino acid for lymphocyte proliferation, to kynurenine (Kyn), highly toxic for effector cells. This enzyme exists in two isoforms (IDO-1, IDO-2). IDO-1 is mainly responsible for tryptophan degradation and highly expressed in multiple types of human cancer, including acute myeloid leukemia (69, 70). Although IDO-2 is expressed in some human tumors, its function still remains to be clarified (71). IDO-1 can be induced in immune cells recruited by the tumor, especially APCs through canonical and non-canonical pathways including NF-κB, Jak/STAT, PKC and TGF-β signaling pathways (72). The rise in the Kyn/Trp ratio in cancer patients suggested an increase in IDO activity and low concentrations of tryptophan in serum/plasma is a reflection of the chronic activation of IDO-1 in the TME, which correlates with tumor progression and poor patient outcomes (73). The Trp shortage, which results in mTORC1 inhibition and general control non-derepressible 2 (GCN2) activation, leads to an anergic status of effector T cells (72). IDO-1 also reduces cytokine release, favors the expansion of Tregs and MDSCs, (74), regulates the differentiation of tolerogenic DCs (75), and along with PGE2, mediates the inhibitory effect of major NK receptors (NCRs and NKG2D), creating a consequent impairment of NK cell-mediated cytolytic activity (76). Using a mouse pregnancy model, IDO-1 highly expressed in the placenta, or products of tryptophan catabolism play a role in maternal T-cell activity suppression, hence protecting the mouse fetus from the maternal immune rejection (77). This effect of rejection was observed in the context of cancer. The expression of IDO by immunogenic murine tumor cells prevents their rejection by preimmunized mice due to a lack of specific T cell accumulation at the tumor site. This can be reverted by systemic treatment of mice with an inhibitor of IDO (78). These preclinical data led to a rapid clinical development of the first generation IDO-1 inhibitors (indoximod, 1-MT, NLG8189), which has been demonstrated to relieve IDO-mediated immunosuppression *in vitro* and *in vivo* by the creation of a critical Trp-sufficiency signal that bypasses activation of GCN2 and inhibition of mTOR in conditions of Trp deprivation (79).

The US FDA has approved the clinical registration applications of IDO inhibitors, in 2016, PF-06840003 in the completed trial NCT02764151; in 2017, NLG802 in NCT03164603 and HTI-1090, respectively, in NCT03208959. Furthermore, combinational regimens with other treatment modalities are under evaluation (80), and some of them such as the phase I/II trial of indoximod combined with temozolomide, have shown promising results in patients with primary malignant brain tumors (NCT02052648). The completion of the safety and efficacy study evaluating indoximod in combination with gemcitabine and nab-paclitaxel in patients with metastatic pancreatic cancer (NCT02077881) revealed a promising activity as shown by the increased in intratumoral CD8+ T-cell density (poster presentation at the annual meeting of the American Society of Clinical Oncology 2018). In a phase II trial, indoximod plus ICB in patients with advanced melanoma (NCT02073123) showed a favorable overall response rate compared to pembrolizumab alone. The combination of the potent and selective oral inhibitor of IDO-1, epacadostat (INCB024360) with nivolumab demonstrated safety, tolerability, and efficacy for treatment of patients with naïve advanced melanoma and head and neck squamous cell carcinoma (HNSCC) in a phase I/II trial (ECHO-204, NCT02327078). Another phase I/II trial evaluating epacadostat plus pembrolizumab (ECHO-202, NCT02178722) demonstrated activity in patients with advanced NSCLC. Though in a phase III clinical trial, epacadostat plus pembrolizumab did not meet the primary endpoint of improving PFS in patients with unresectable or metastatic melanoma when compared to pembrolizumab monotherapy (ECHO-301/KEYNOTE-252, NCT02752074). Despite some promising results, concerns have been raised regarding the use of IDO inhibitors which can result in severe autoimmune reactions. Indeed, as IDO can be activated by many stimuli such as IFN-γ and tumor necrosis factor (TNF)-α, IDO inhibitors may not work in a patient with a cold tumor, not infiltrated by T cells.

### Arginase and Inducible Nitric Oxide Synthase (iNOS)

The semi-essential amino acid arginine, also known as L-arginine is a precursor for several metabolites and a critical regulator of lymphocyte proliferation and function. The arginases (Arg1 and 2) and nitric oxide synthetases (NOS1-3) are the major enzymes responsible for arginine metabolism in inflammatory immune responses (81, 82). Arg1 and 2 induce the same reaction but differ in tissue distribution and intracellular localization (83). Tumor arginase deprives immune cells within the local TME of arginine by catalyzing the hydrolytic conversion of arginine into L-ornithine and urea, resulting in dysfunctional immune cells (81, 84). This degradation of extracellular arginine affects CD3 ζ chain, the main signaling chain of the TCR, resulting in T cell anergy (84). The depletion of L-arginine also induces a blockade in infiltrating T-cells and cell cycle progression (81). The immunosuppressive enzyme from the NOS family, NOS2 (inducible NOS or iNOS) is inducible by inflammatory cytokines and metabolizes L-arginine to produce reactive free radical NO and L-citrulline (81). The cellular signaling molecule NO was found to be actively associated with tumors as well as the tumor environment and in addition to its role in cancer initiation and progression, NO contributes to the anti-tumor immune response and limits T cell proliferation and activity by promoting apoptosis and by inhibiting cytokine and chemokine production (85, 86). NOS2 is expressed by various cell types involved in inflammation, including neutrophils, M2 macrophages, MDSCs, DCs, NK cells, endothelial and tumor cells (87). Upregulation of arginase prevents NOS2 activity, whereas arginase inhibition causes enhanced NOS2 expression and leads to increased NO production. TAMs and MDSCs overexpress and secrete Arg1 and NOS2 to deplete intracellular and extracellular arginine facilitating the T cell impairment. Moreover, in MDSCs, the expression of ARG1 and iNOS is regulated by cyclic GMP levels, which is in turn controlled by the activity of phosphodiesterase type 5 (PDE5). Therefore, the agents that can elevate intracellular cGMP levels, such as PDE5 inhibitors, reduce MDSC-mediated immune suppression (88).

Decreased circulating arginine levels in cancer patients are considered indicative of elevated plasma levels and high Arg1 expression in tumors (89). Up to date, only a few studies have been conducted with arginase inhibitors in different human cancers, mostly to avoid complications related to the conversion of highly toxic ammonia to urea which will be excreted. However, in late 2017, the first cohort of patients with advanced/metastatic solid tumors was treated with INCB01158 (an Arg1 inhibitor formerly known as CB-1158) monotherapy or combined with pembrolizumab (NCT02903914). PDE-5, initially used for the treatment of erectile dysfunction, brings clinical benefits in a range of cancers by acting on the NO/cyclic guanosine monophosphate (cGMP) signaling pathway (90). Tadalafil, a PDE-5 inhibitor decreases circulating MDSCs, lowers iNOS and arginase expression in these cells, and enriches tumor-specific T cells in HNSCC patients (91) and it is being clinically tested in patients with multiple myeloma in combination with lenalidomide (NCT01858558) and in patients with HNSCC in combination with mucin 1 vaccine (NCT02544880).

### Ectonucleotidases CD39 and CD73

The accumulation of adenosine, an extracellular immunosuppressive metabolite, is a strategy used by tumors to evade immunosurveillance. Extracellular adenosine triphosphate (ATP) and adenosine are abundant metabolites and have an important autocrine/paracrine role. ATP is catabolized to adenosine in the TME by two ectonucleotidases: CD39 and CD73, anchored in cancer cells, regulatory immune and endothelial cells from the vasculature (88). CD39 and CD73 can be recognized as “immune checkpoint mediators” since they interfere with anti-tumor immune responses (92). CD39 reversibly produces AMP from ATP or ADP, which is subsequently converted into extracellular adenosine by CD73 (93). The hypoxic TME maintains elevated levels of adenosine due to the high-level expression of CD73 by tumor cells, which results in a chronic suppression of immune cells (88). In addition to tumor cells, adenosine can also be the product of immune cells. CD56^bright^ NK cells release adenosine in the presence of autologous CD4^+^ T cells (94). Tregs overexpress CD39 and CD73 responsible of the sequential conversion of pro-inflammatory extracellular ATP into AMP and adenosine (95). Among the four adenosine-binding G protein-coupled receptors, A2A receptor is the most expressed subtype on immune cells: T, NK, NKT, macrophages, and DCs (96). Upregulation and activation of A2A receptor switches macrophages from an M1 to an M2 phenotype and induces the production of VEGF and IL-10 (97). When binding to A2B receptor, adenosine promotes the expansion and functions of MDSCs (98), and induces high expression levels of angiogenic, proinflammatory, immune suppressor, and tolerogenic factors (99). Using an A2aR-null mouse model, several melanoma and T cell lymphoma lines were rejected in a CD8^+^T cell dependent manner, and addition of a pharmacologic blockade of A2aR could enhance T cell mediated tumor regression in a sarcoma and LL-LCMV tumor model (100). Other preclinical studies have focused on blocking the adenosine pathway by targeting CD73 and/or CD39. CD73- null mice significantly induce tumor rejection in a variety of syngeneic tumor models (101) and, CD39-null mice were resistant to tumor metastases in B16/F10 mouse melanoma and MCA-38 colorectal models (102). In addition, weakening of upstream tumor hypoxia by supplemental oxygenation decreases the intensity of downstream A2AR-mediated immunosuppression in mice (103). Co-inhibition of CD73 and A2ARs in mice with spontaneous or transplantable tumors improve the inhibition of tumor initiation, growth, and metastasis (104).

It has been reported that CD39-expressing-melanoma cells inhibit both T cell proliferation and generation of cytotoxic effectors in an adenosine-dependent manner (105). Recently, in a phase I/Ib clinical trial CPI-444, an oral small molecule targeting the adenosine-A2A receptor combined with the intravenous PD-L1 inhibitor atezolizumab demonstrated an OS of 88% at more than 20 months follow-up in treatment-refractory RCC patients (NCT02655822). Another agent targeting A2A receptor, NIR178 (PBF-509) in combination with an anti-PD1 is currently under investigation for the treatment of advanced NSCLC. Clinical benefits were reported in immunotherapy-exposed and -naïve patients irrespective of PD-L1 status (NCT02403193).

## Co-inhibitory Molecules

### Cytotoxic T-Lymphocyte Antigen-4 (CTLA-4)

Member of the immunoglobulin superfamily, CTLA-4 (CD152) was the first discovered co-inhibitory receptor (106). CTLA-4 shares the same ligands with CD28, namely CD80 (B7.1) and CD86 (B7.2) but with less affinity, thus counteracting the stimulatory effects of CD28 ligation (107). Mice deficient in CTLA-4 develop generalized lymphoproliferative syndrome with a lymphocytic infiltration of all organs (108). Using mice bearing partially immunogenic tumors, the Allison group showed that CTLA4 blockade could enhance the endogenous anti-tumor response after tumor implantation (109). CTLA-4 was then clinically targeted, and the following relevant investigations of its role in modulation of the amplitude of the early stages of T cell activation represent the breakthrough of cancer immunology (110). The proposed mechanism is the direct inhibition at the TCR immune synapse, inhibition of CD28 or its signaling pathway, or increase in mobility of T cells that are less prone to interact with APCs (111). CTLA-4 is expressed by effector T cells and constitutively expressed by Tregs (112). To prevent T cell activation, Tregs primarily target APC via the engagement of CTL-4 with CD80 and/or expression on APCs and transmitting inhibitory signals (113). The mobilization of CTLA-4 from the intracellular protein stores to the cell surface happen as early as an hour after antigen engagement, thus allowing the occurrence of a feedback inhibition. The balance of CD28 and CTLA-4-derived signals is critical for maintaining the equilibrium between T cell activation or tolerance. When administered to patients, mAbs that block the binding of CTLA-4 to its ligands were able to unleash antitumor responses (114).

A pivotal phase III clinical trial launched in 2010 showed that ipilimumab, a fully humanized anti-CTLA-4 mAb, alone or in combination with gp100 peptide vaccine, improved survival in metastatic melanoma patients (115). Ipilimumab was the first FDA-approved checkpoint immunotherapy in patients with advanced melanoma. Afterwards, this agent was approved by the European Medicines Agency (EMA) and is currently indicated for the first line treatment of melanoma and in adjuvant settings.

### Programmed Death 1 (PD-1)/PD-L

PD-1 (CD279) negatively regulates the activity of effector T cell within tissues and tumors, where the immune response is already ongoing, while CTLA-4 is expressed only expressed in T and pro-B cells, the member of the immunoglobulin superfamily is more broadly expressed. (116). PD-1 can bind two ligands from the B7 protein family: PD-L1 (B7-H1, CD274) expressed by macrophages, DCs, activated T and B cells, tumor cells, and tissues, such as heart, lung, spleen and PD-L2 (B7-DC, CD273), which is mainly expressed on DCs and tumor tissues. Unlike CTLA-4, PD-1 acts in the secondary immune response and its expression on the surface of activated T cells is delayed (6–12 h) due to the need of transcriptional activation. PD-1 binds to its ligands directly overexpressed on cancer cells, clusters with TCR and recruits the inhibitory phosphatase SHP2 (Src homology 2 domain-containing tyrosine phosphatase 2) via its immunoreceptor tyrosine inhibitory motif, which induces dephosphorylation of the proximal TCR signaling molecules, thus suppressing T cell activation (117). High levels of PD-1 expression on T cells induces a state of anergy and exhaustion, as shown by *in vitro* and *in vivo* models (118). PD-L1 is expressed by different tumor types (breast, ovary and colon carcinomas), and its expression is up-regulated in the presence of IFN-γ that is released in the TME. The binding of PD-1 to PD-L1 generates an immunosuppressive effect, impairs T cell activation (119) and in addition, increases the proliferation of the tumor infiltrating Tregs (120). A preclinical study in glioblastoma showed that targeting factors responsible for the myeloid PD-L1 upregulation, such as IL-6 enhance the anti-tumor activity exerted by PD-1 therapy. Thus, interfering with IL-6 signaling diminished myeloid immunosuppression, tumor growth, and increase mice survival (121).

In 2014, nivolumab (OPDIVO, Bristol-Myers Squibb Company), an anti-PD-1 mAb was approved by the FDA for melanoma patients and marked the start of several other approvals in other cancer types. In 2015, the FDA approved nivolumab for the management of advanced metastatic RCC after progression on first-line therapy or following prior anti-angiogenic therapy (approval based on an extension in OS in the CheckMate-025 trial, NCT668784), squamous NSCLC as a second-line treatment across all histologies (approval based on data from the phase III CheckMate-057 trial, NCT01673867), advanced or metastatic urothelial carcinoma (phase II CheckMate-275 trial, NCT02387996) and hepatocellular carcinoma after sorafenib treatment (CheckMate 040 trial, NCT01658878). Nivolumab became the first and only immuno-oncology treatment option for patients with metastatic small cell lung cancer progressed after platinum-based chemotherapy and at least one other line of therapy. Interestingly, in the adjuvant setting, results from the phase III, NCT02388906 (CheckMate 238) comparing the efficacy of nivolumab vs. ipilimumab in patients with resected stage III/IV melanoma at high risk of recurrence showed that nivolumab provides superior safety and survival compared to ipilimumab, regardless of tumor PD-L1 status. Several trials combined nivolumab with other cancer therapies. Combination with gemcitabine, which induces the killing of MDSCs, increases the efficacy of nivolumab in metastatic NSCLC (NCT03302247). The trial NCT02922764 is evaluating the combination of RGX-104, agonist of the nuclear receptor liver X receptor (LXR) with nivolumab in advanced solid malignancies and lymphoma. This interaction between LXR and RGX-104 induces depletion of both MDSCs and endothelial cells. The clinical study NCT01454102 (CheckMate 012) testing the combination of nivolumab plus ipilimumab as first line treatment for advanced NSCLC patients showed durable efficacy and resulted in a significantly longer OS than ipilimumab alone in a phase III trial involving patients with advanced melanoma [CheckMate 067, NCT01844505, (122)]. The trial NCT01472081 (CheckMate 016) evaluated the safety and efficacy of nivolumab combined with ipilimumab in patients with metastatic RCC (123). And in April 2018, nivolumab has been approved in combination with ipilimumab as first-line treatment for patients with advanced RCC. Another anti-PD-1 agent, pembrolizumab (KEYTRUDA, Merck & Co., Inc.) was approved for advanced melanoma in 2014, followed by several other approvals in other cancer types, such as advanced NSCLC in 2015, Hodgkin lymphoma and urothelial carcinoma in 2017. Interestingly, in May 2017, for the first time, the FDA approved a cancer drug based on tumor genetics rather than tissue type or tumor site. Indeed, pembrolizumab was granted approval for the treatment of any unresectable or metastatic solid tumor with specific genetic features (mismatch repair deficiency or microsatellite instability). In 2018, the FDA granted the approval of pembrolizumab for treating recurrent or metastatic cervical cancer whose tumor expresses PD-L1 and refractory or relapsed mediastinal large B-cell lymphoma. The clinical trial identified as NCT03241927 is currently investigating the effect of pembrolizumab on NK cell exhaustion in melanoma based on the idea that releasing the PD-1 immune checkpoint in NK cells may help them to infiltrate the tumor and exert their effector functions against the tumor. Results from the phase III study (NCT02252042) assessing the antitumor activity and toxicity of pembrolizumab in patients with recurrent or metastatic HNSCC showed a lower risk of death in patients with high tumor PD-L1 expression, and this despite missing predetermined endpoints. The combination of the histone deacetylase inhibitor entinostat with pembrolizumab is currently under investigation in patients with advanced solid tumors (NCT02909452), and so far, resulted in a reduction in monocytic MDSCs across all the treatment arms. Hypothetically, treating patients resistant to anti-PD-1 with a TLR agonist injected into the tumor may allow the TME to be more immunogenic and thus, more sensitive to PD-1 inhibition. In this context, pembrolizumab combined with CPM-001, an agent that activates TLR-9 is being tested in patients with advanced melanoma resistant to PD-1 inhibition (NCT02680184). Reports are showing objective and durable tumor responses. A phase I/II study combining pembrolizumab with targeted molecule inhibitors (BRAF plus MEK) is ongoing in melanoma and other solid tumor patients (NCT02130466). Other clinical studies are evaluating the efficacy of pembrolizumab plus chemotherapy (cisplatin, capecitabine or 5-fluorouracil) (NCT02494583). Recently, the FDA granted approval for pembrolizumab in combination with chemotherapy (pemetrexed and platinum) for first line treatment of metastatic non-squamous NSCLC, with no EGFR or ALK genomic tumor aberrations. Several anti-PD-L1 mAbs are under investigation in various cancers including melanoma, multiple myeloma, leukemia, lymphoma, glioblastoma as well as gastric, renal cell, bladder, colorectal, hepatocellular, cutaneous, breast and NSCLC cancers. Atezolizumab (Tecentriq, Genentech) is currently being tested in combination with bevacizumab and/or with chemotherapy in patients with locally advanced or metastatic solid tumors (NCT01633970). In 2016, the FDA approved atezolizumab as first line treatment for cisplatin resistant metastatic urothelial carcinoma and metastatic NSCLCs. In April 2017, atezolizumab became the first cancer immunotherapy approved by the FDA for patients with advanced bladder cancer and thus is employed as a standard of care. Avelumab (Bavencio, EMD Serono, Inc.) is a fully human anti-PD-L1 mAb which received FDA approvals in March and May 2017 for the treatment of patients with metastatic Merkel-cell carcinoma and advanced or metastatic urothelial carcinoma during or after treatment with platinum-containing chemotherapy administered in neoadjuvant or adjuvant setting. Recently discovered through a novel genome-scale T cell activity array, the immune suppressor Siglec-15 which is highly expressed on tumor cells and tumor-infiltrating myeloid cells can serve as a biomarker for predicting the outcomes of anti-PD-1/PD-L1 therapy. Siglec-15 has been shown to continuously inhibit T cell activity. Thus, using anti-Siglec-15 therapy may offer an alternative strategy to PD-1/PD-L1 pathway and lead to tumor immune normalization (124).

### Lymphocyte-Activated Gene-3 (LAG-3, CD223)

LAG-3 is a surface molecule structurally related to CD4 (125). LAG-3 consists of four extracellular immunoglobulin superfamily-like domains (D1-D4) and binds to MHC class II molecules with greater affinity than CD4, utilizing an additional 30 amino acid loop (126). Like the CTLA-4/CD28 subfamily, the LAG-3/CD4 subfamily represents an inhibitory/stimulatory receptor subfamily modulating TCR signaling. LAG-3 is expressed in activated CD4^+^ and CD8^+^ T cells 3–4 days post activation (127), on a subset of NK cells (128) and on activated Tregs (129). LAG-3 does not feature an immunoreceptor tyrosine-based inhibition motif (ITIM) but possesses in its cytoplasmic tail two distinct motifs mediating the intrinsic negative inhibitory signal: a repetitive “EP” motif consisting of a series of glutamic acid-proline dipeptide repeats, and a single lysine residue (K468) within the conserved KIEELE motif in the cytoplasmic domain (130). The interaction between highly constitutive expression of MHC class II molecules at the surface of melanoma cells and LAG-3 greatly expressed on melanoma-specific CD4^+^ T cells elicits a local TNF-rich inflammatory environment, reducing the cytotoxic CD8^+^ T cell responses (131). LSECtin, expressed on melanoma is a type II transmembrane protein which belongs to the C-type lectin receptor superfamily and has been identified as a ligand of LAG-3. When LSECtin interact with the co-regulatory molecule LAG-3, and limits CD8^+^ T cell- specific responses in a LAG-3-dependent fashion (132). Galectin-3, an S-type lectins is a carbohydrate-binding protein that plays a key role in tumor escape from immunosurveillance (133, 134). Galectin-3-mediated suppression of CD8^+^T cells occurs upon binding to LAG-3 (135). Interestingly, co-expression of LAG3 with CD49b defines a subset of peripherally induced CD4^+^ Th1 regulatory cells secreting high amounts of IL-10 (136), and it is also involved in the maturation and activation of DCs (137) and plasmacytoid DCs (138). Recently, fibrinogen-like protein 1 (FGL1) has been shown to be a key ligand of LAG-3. Abundantly produced by cancer cells, FGL1 plasma level is increased in cancer patients and correlates with poor prognosis (139). Preclinical studies exploring the immune regulatory role of LAG-3 on various types of lymphocytes have showed its cooperation with other inhibitory receptors, such as PD-1/PD-L1. The association of LAG-3 and PD1 contributes to their rapid trafficking to the immunological synapse, leading to the synergistic inhibitory effect on T cell signaling in several tumor murine models (140, 141). In addition, LAG-3 and PD-1 are co-expressed on tumor infiltrating lymphocytes (TIL)-specific CD8^+^ T cells in the peripheral blood and tumors of ovarian cancer patients resulting in T cell dysfunction (142).

LAG-3-targeted immunotherapy started in 2006 with a toxicology study aiming to determine the dosage and frequency of the preclinical grade human soluble LAG-3 protein named IMP321 (143). In 2013, a phase I clinical trial with the anti-LAG3 mAb (BMS-986016) was initiated. There are now several LAG3 modulators at various stages of preclinical and clinical development for the treatment of relapsed or refractory hematologic malignancies (NCT02061761), and advanced cancers, alone or in combination with anti-PD1 in patients with (NCT02966548, NCT03005782). A phase I/IIa study (NCT01968109) evaluating efficacy and safety of the combination therapy with the anti-LAG-3 antibody BMS-986016 (relatlimab) and nivolumab in patients with melanoma that progressed during or after anti-PD-1/PD-L1 immunotherapy has reported a safety profile similar to that of nivolumab alone, but with superior efficacy.

### T Cell Immunoglobulin and Mucin 3 (TIM-3)

TIM-3 is a surface negative regulator of CD4+ Th1, CD8+ T cytotoxic 1 cells, and innate immune cells and contain an immunoglobulin and a mucin-like domain (144, 145). TIM-3 regulates Th1 and Th17 responses when interacting with its ligands, thus inhibiting the expression of proinflammatory cytokines such as INF-γ and TNF-α (145, 146). Galectin-9 (Gal-9), expressed in various tumors has a key role in tumor immunity (147). Gal-9 was the first TIM-3 identified ligand and binds the Tim-3 immunoglobulin variable domain to regulate Th1 immunity in TIM-3-deficient mice (148). Other identified ligands of TIM-3 include: carcinoembryonic antigen cell adhesion molecule 1 (CEACAM-1), high-mobility group protein B1 (HMGB1), and phosphatidylserine (PS) (149). TIM-3 contain two of its more membrane-proximal cytoplasmic tail tyrosines which can directly bind to the Src family tyrosine kinase Fyn and the PI3K adaptor but no any known inhibitory signaling motifs. Expressed on tumor-infiltrating DCs, TIM-3 plays a critical role in suppressing innate antitumor immune responses through the recognition of tumor-derived nucleic acids (150). Interestingly, the inhibition of the antitumor responses via TIM-3 mediates T cell exhaustion, a phenomenon that was first identified *in vitro* in patients with HIV-1 infection (151), and later in cancer patients (152).

In RCC, TIM-3 expression has been shown to be elevated on tumor and myeloid cells of patients (153), and its upregulation on CD8^+^T cells lead to immune evasion at relatively early stage (154). Furthermore, prostate-specific antigen cells expressing high levels of TIM-3 are exhausted, influencing a patient's response to therapy (155). In patients with advanced melanoma, a majority of CD8^+^ TILs co-express PD-1 and TIM-3 (156). The co-expression of both checkpoint molecules reflected a more exhausted phenotype characterized by reduced T cell proliferation and IFN-γ, IL-2, and TNF-α secretion (116). In addition, overexpression of TIM-3 can function as an NK-cell exhaustion marker in advanced melanoma and is associated with a poor prognosis (157). A phase I study is evaluating TSR-022, an anti-human TIM-3 blocking antibody as monotherapy and in combination with an anti-PD-1 antibody for patients with advanced solid tumors who had limited available treatment options (NCT02817633). The safety and effectiveness of the anti-TIM-3 mAb (MBG453) is being evaluated in patients with solid tumors, either alone or combined with another immunotherapy (NCT02608268) and the combination MBG453-decitabine in patients with hematologic cancers (NCT03066648). The clinical results are not yet reported.

### V-Domain Ig-Containing Suppressor of T Cell Activation (VISTA)

VISTA, also known as PD1 homolog (PD-1H), belongs to the B7 family members. VISTA contains a single IgV domain with three additional cysteine residues, which differs structurally from the other B7 family members (158). VISTA is known to play a central role in the regulation of T cell responses and its extracellular domain has some similarities with PD-L1 (159). While VISTA does not contain ITIM/ immunoreceptor tyrosine-based activation motif (ITAM), it has a conserved Src homology 2 (SH2) and its cytoplasmic tail domain contains two potential protein kinase C binding sites and proline residues that could function as docking sites for adaptor proteins. VISTA transcription is partially controlled by p53 (160). VISTA has been shown to have a dual functionality as a co-inhibitory receptor on T cells (161) and as a co-inhibitory ligand for T cells (158, 159). Recently reported, blocking the interaction of VISTA with its ligand VSIG-3 inhibits human T cell functions *in vitro*, as well as cytokine and chemokine production in colorectal, gastric cancers, and hepatocellular carcinomas (162). In humans and mice, VISTA is expressed mainly on hematopoietic cells with high levels on myeloid APCs, a weak density on T cells and NK cells, and no expression on B cells (158, 159, 163). In addition to immune cells, it has been demonstrated that high levels of VISTA are also expressed on human and murine tumor cells (158, 164). Anti-VISTA monotherapy with an mAb significantly reduces the growth of the tumor in multiple transplantable or inducible tumor models of melanoma and bladder carcinoma (163). Furthermore, combining blockades of VISTA together with CTLA-4 is more efficient than the PD-1 and VISTA combination in the HNSCC model (165). While in primary cutaneous melanoma patients, VISTA expression is considered as an independent negative prognostic factor (166), its overexpression on immune cells, especially macrophages, that infiltrated pancreatic tumors has been highlighted as a potential immunotherapeutic target (167). VISTA upregulation in prostate cancer may represent a compensatory inhibitory pathway after ipilimumab administration (168).

Blocking the interactions between PD-1/PD-L1 and VISTA using CA-170, a small molecule that antagonizes the PD-L1/PD-L2 and VISTA pathways improves the anti-tumor responses in certain tumor models and highlights their distinct and non-redundant functions in regulating the immune response to tumors (169). Preclinical data demonstrated VISTA's ability to independently suppress T cell responses, thus supporting the starting of a phase I dose escalation trial in patients with advanced tumors and lymphomas in 2016 (NCT02812875). So far, in toxicology studies, CA-170 showed a favorable safety profile when multiple dose levels were administered orally once daily, and preliminary evidence of antitumor activity. Interestingly, a recent immunotherapy clinical trial using CA-170 has been launched in mesothelioma patients. Currently, CA-170 is the only human mAb against VISTA being studied in a clinical trial in advanced cancers, since the recruitment for the trial NCT02671955 evaluating JNJ-61610588 (Onvatilimab) has been stopped due to a business decision done by Janssen.

### T Cell Immunoglobulin and Immunoreceptor Tyrosine-Based Inhibitory Domain (TIGIT)

TIGIT (also known as WUCAM, Vstm3, VSIG9) is a type 1 transmembrane protein containing an IgV extracellular domain and two ITIMs in its cytoplasmic tail. These motifs mediate the recruitment of the phosphatase SHIP-1, thus providing a mechanism by which TIGIT can dampen activating signals (170). Engagement of CD155 (poliovirus receptor, PVR) ligand with the co-inhibitory receptor TIGIT leads to T cell inhibition responses, while the interaction of CD155 with the co-stimulatory receptor CD226 (DNAM-1) leads to T cell activation (171). TIGIT is associated with human cancers and expressed on NK and lymphoid cell populations (activated and memory T cells, and a subset of Treg). This provide an opportunity to target both the adaptive and innate arms of the immune system (172, 173). In murine cancer models, TIGIT^+^ Tregs may drive a dysfunctional phenotype in CD8^+^T cells via their high production of IL-10 (174). Interestingly, due to cellular stress within the TME, the expression of CD155 increase at the surface of APCs during the malignant transformation (175). Moreover, the interaction between TIGIT and CD155 on mature DCs induces a switch into a tolerogenic phenotype in DCs (171). TIGIT deficiency in NK cells alone has been reported to be sufficient to delay tumor growth in multiple tumor-bearing mouse models, and anti-TIGIT mAbs reverse NK cell exhaustion (176). Other preclinical data demonstrated that anti-TIGIT treatment reduced the abundance of Tregs within tumors in animal models (Abstract 5627, AACR annual Meeting 2018).

Preclinical studies demonstrated its efficacy in *in vivo* tumor models and higher effectiveness when combined with other checkpoint inhibitors. Initial evaluation of the anti-TIGIT mAb, OMP-31M32 (Etigilimab) in a phase Ia/b showed preclinical *in vivo* anti-tumor effects as a single agent and in combination with anti-PD-1 (NCT03119428) results presented at the Society for Immunotherapy of Cancer 2018). In an ongoing phase I/II trial, the experimental medication BMS-986207 is evaluated for its safety and effectiveness as monotherapy or in combination with nivolumab for the treatment of advanced or metastatic solid cancers (NCT02913313). Soon, EOS884448, an anti-TIGIT antibody disrupting the immunosuppressive binding of CD155 to TIGIT in the TME and mediating the restoration of T cell effector functions and the preferentially depleting Tregs is expected to enter the clinic.

**Figure 1:**
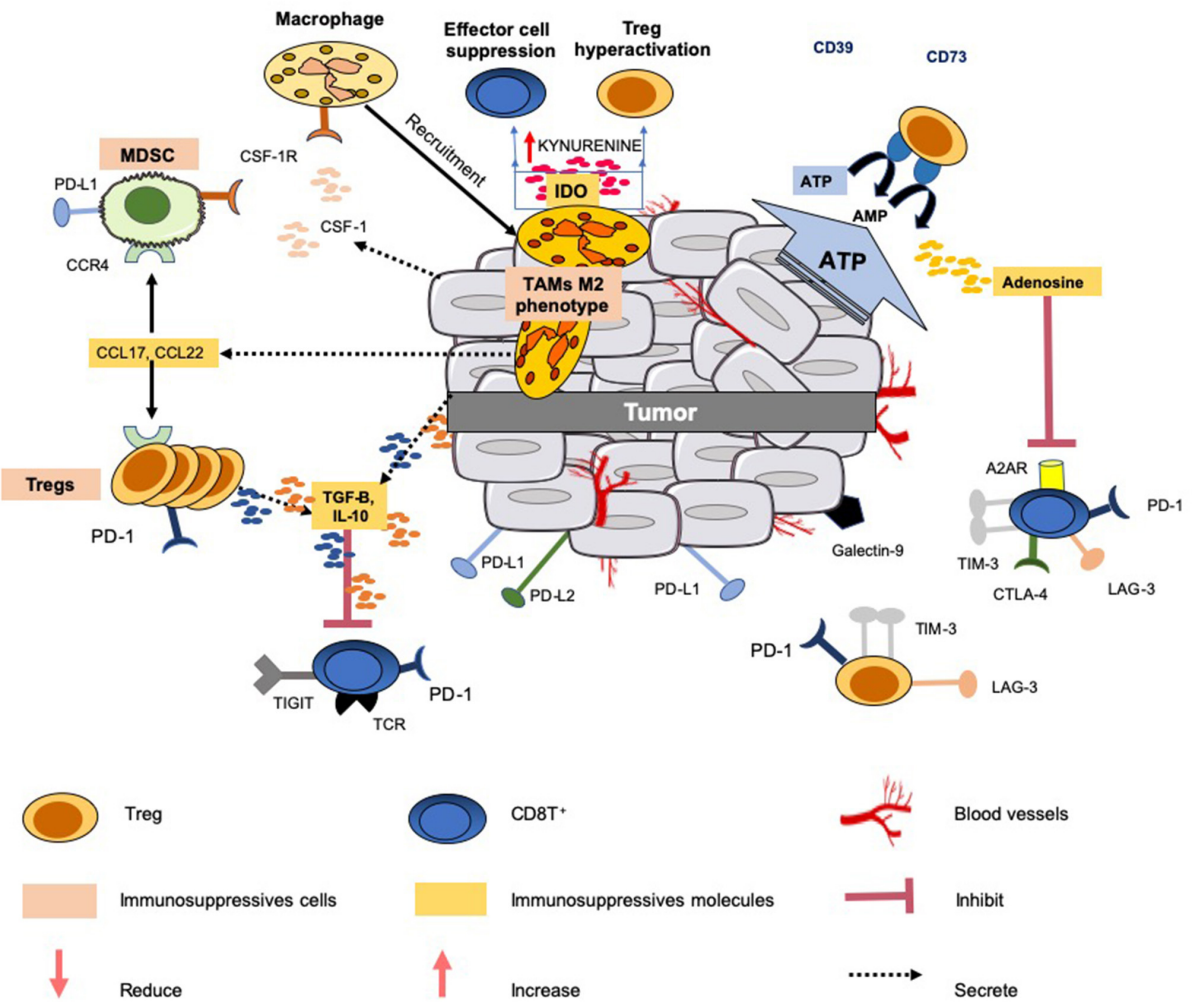
Immunosuppressive mechanisms common in the tumor microenvironment. Several mechanisms are developed by the tumor to limit an efficient tumor immunosurveillance, and therefore an unfavorable environment. This explains that a portion of epithelial cancers displays modest responses to immune checkpoint blockade therapy and other modulators of immunity. All the mechanisms known to interfere with these immunotherapies do not operate simultaneously in these cancers. This figure illustrates only a few common mechanisms of immune resistance to different tumors: (1) production and secretion of immunosuppressive factors into the microenvironment (such as TGF-β, IL-10, adenosine); (2) co-expression and/or upregulation of inhibitory receptors (LAG3, TIGIT, TIM-3,TIGIT) by immunosuppressive cells (Tregs, MSDCs and TAMs) and effector cells (CD8T^+^ and NK); (3) release of the chemokines CCL17 and CCL22 by the tumor which triggers the accumulation of Tregs and MDSCs to tumor sites; (4) release of IL-10 and TGF-β by Tregs which inhibit the functions of CD8^+^ T cells; (5) IDO expression by TAMs metabolizes tryptophan to kynurenine and limits T-cell function. Additionally, the tumor can also gain additional immunosuppressive properties, such as the expression of PD-L1, PD-L2, and secretion of suppressive cytokines (e.g., IL-10, TGF-β).

The main immunological abnormalities conferring tumor evasion are illustrated in Figure 1. Some of the ongoing clinical trials aiming to assess the role of soluble factor inhibitors, enzymes and metabolic inhibitors and immune checkpoint inhibitors alone or in various combination with other cancer therapeutics are summarized in the Tables 1–3.

**Table 1:** Clinical studies testing agents targeting soluble factors, enzymes and metabolic inhibitors.

**Inhibition**	**Study ID number**	**Drug name**	**Indication(s) (cancer type/subtype)**	**Setting**	**Stage of development and status**
IL-10 pathway	NCT03382899	AM0010 (pegilodecakin)	Metastatic non-small-cell lung carcinoma	Combination: • Pembrolizumab	Phase II, recruiting
	NCT02923921	AM0010	Metastatic pancreatic cancers	Combination: • FOLFOX (Folinic acid+ 5- Fluorouracil+Oxaliplatin)	Phase III, recruiting
VEGF pathway	NCT02443324	Ramucirumab (anti-VEGFR2)	Locally advanced and unresectable or metastatic gastric or gastroesophageal junction adenocarcinoma, non-small cell lung cancer, transitional cell carcinoma of the urothelium, or biliary tract cancer	Combination: • Pembrolizumab	Phase I, active not recruiting
	NCT02348008	Bevacizumab	Metastatic renal cell carcinoma	Combination: • Pembrolizumab (MK-3475)	Phase Ib/II, active not recruiting
	NCT01633970	Bevacizumab	Advanced or metastatic solid tumors	Combination • Atezolizumab • Atezolizumab+Oxaliplatin+5- Fluorouracil	Phase I, active not recruiting
	NCT01454102 (Checkmate 012)	Bevacizumab maintenance	Newly diagnosed or pretreated stage IIIB/IV NSCLC	Combination: • Nivolumab	Phase I, active not recruiting
	NCT02231749 (CheckMate 214)	Sunitinib	Advanced or metastatic renal cell carcinoma	Monotherapy	Phase III, active not recruiting
CSF-1 pathway	NCT03336216	Cabiralizumab (anti-CSF-1R)	Advanced pancreatic cancer	Combination • Nivolumab + gemcitabine and Nab-paclitaxel • Nivolumab + oxaliplatin/5-Fluorouracil/leucovorin	Phase II, recruiting
TGF-β pathway	NCT02517398	MSB0011359C (M7824) bifunctional fusion protein (PD-L1 fused to the soluble extracellular domain of TGF-β receptor II)	Metastatic or locally advanced solid tumors	Monotherapy	Phase I, recruiting
	NCT02423343	Galunisertib	Solid tumors, non-small cell lung cancer recurrent, hepatocellular carcinoma recurrent	Combination • Nivolumab	Phase I/II, active not recruiting
	NCT02734160	Galunisertib	Metastatic pancreatic cancer	Combination • Durvalumab	Phase I, active not recruiting
	NCT02672475	Galunisertib	Triple negative breast cancer	Combination • Palcitaxel	Phase I, recruiting
IDO	NCT03164603	NLG802	Advanced solid tumors	Monotherapy	Phase I, active not recruiting
	NCT03208959	HTI-1090	Advanced solid tumors	Monotherapy	Phase I, active not recruiting
	NCT02052648	Indoximod	Primary malignant brain tumors	Combination: • Temozolomide (bevacizumab-naive patients) • Temozolomide with bevacizumab • Temozolomide with stereotactic radiation	Phase I /II, active not recruiting
	NCT02073123	Indoximod	Metastatic melanoma	Combination with checkpoint inhibitors (ipilimumab or nivolumab or pembrolizumab)	Phase I, active not recruiting
	NCT02327078 (ECHO-204)	Epacadostat	Selected solid tumors and lymphoma	Combination: • Nivolumab • Nivolumab + chemotherapy	Phase I /II, active not recruiting
	NCT02178722 (ECHO-202)	Epacadostat	Selected cancers	Combination: Pembrolizumab (MK-3475)	Phase I /II, active not recruiting
	NCT02752074 (ECHO-301, KEYNOTE-252)	Epacadostat	Unresectable or metastatic melanoma	Combination • Pembrolizumab	Phase III, active not recruiting
Arg/iNOS	NCT02903914	INCB001158 (CB-1158, arginase 1 inhibitor)	Advanced / metastatic solid tumors	Monotherapy Combination: • Pembrolizumab	Phase I /II, recruiting
	NCT01858558	Tadalafil (PDE5 inhibitor)	Multiple myeloma patients who receive a standard autologous stem cell transplant	Combination: Pevnar vaccine (pneumococcal 7-valent conjugate) + Lenalidomide, with or without activated marrow infiltrating lymphocytes (MILs)	Phase II, recruiting
	NCT02544880	Tadalafil (PDE5 inhibitor)	Head and neck squamous cell carcinoma	Combination: • Anti-Tumor Mucin 1 (MUC1) and anti-influenza vaccine	Phase I /II, recruiting
Adenosine	NCT02655822	CPI-444	Advanced cancers	Monotherapy Combination: • Atezolizumab (anti-PD-L1)	Phase I, recruiting
	NCT02403193	PBF-509	Advanced non-small cell lung cancer	Monotherapy Combination: PDR001 (programmed cell death 1 receptor antibody)	Phase I, recruiting

**Table 2:** Anti-PD-1 and anti-PD-L1 agents in clinical trials as mono- and/or combination therapies.

**Target**	**Study ID number**	**Drug name**	**Indication(s) (cancer type/subtype)**	**Setting**	**Stage of development and status**
PD-1	NCT01668784 (CheckMate 025)	Nivolumab (BMS-936558, MDX-1106)	Advanced or metastatic (medically or surgically unresectable) clear-cell renal cell carcinoma	Monotherapy (Comparison to Everolimus)	Phase III, active, not recruiting
	NCT01673867 (CheckMate 057)	Nivolumab	Metastatic non-squamous NSCLC	Monotherapy Combination: • Docetaxel	Phase III, active, not recruiting
	NCT02387996 (CheckMate 275)	Nivolumab	Urotherial cancer (metastatic or unresectable bladder cancer)	Monotherapy	Phase II, active, not recruiting
	NCT01658878 (CheckMate 040)	Nivolumab	Advanced liver cancer	Monotherapy Combination: • Sorafenib • Ipilimumab • Cabozantinib	Phase I / II, active, not recruiting
	NCT02388906 (CheckMate 238)	Nivolumab	Advanced Melanoma	Monotherapy	Phase III, active, not recruiting
	NCT03302247	Nivolumab	Metastatic non-small cell lung cancer	Monotherapy Combination: • Gemcitabine	Phase II, terminated last June 2019 (unable to accrue subjects)
	NCT02922764	Nivolumab	Advanced solid malignancies and lymphoma	Combination: • RGX-104	Phase I, recruiting
	NCT01454102 (CheckMate 012)	Nivolumab	Non-small cell lung cancer	Monotherapy Combination • Gemcitabine + Cisplatin • Pemetrexed + Cisplatin • Paclitaxel + Carboplatin • Bevacizumab maintenance • Erlotinib • Ipilimumab	Phase I, active, not recruiting
	NCT01844505 (CheckMate 067)	Nivolumab	Untreated advanced melanoma	Monotherapy Combination: • Ipilimumab	Phase III, active, not recruiting
	NCT01472081 (CheckMate 016)	Nivolumab	Metastatic renal cell carcinoma	Combination • Sunitinib (Anti-VEGF) • Pazopanib (Anti-VEGF) • Ipilimumab	Phase I, active, not recruiting
	NCT03241927	Pembrolizumab (MK-3475)	Melanoma	Monotherapy	Phase II, terminated last July 2019, difficult enrollment
	NCT02252042	Pembrolizumab	Head and neck squamous cell cancer	Monotherapy	Phase III, active, not recruiting
	NCT02909452	Pembrolizumab	Advanced solid tumors	Combination: • Entinostat (histone deacetylase inhibitor)	Phase I, active, not recruiting
	NCT02680184	Pembrolizumab	Advanced Melanoma	Combination: CMP-001 (TLR9 activator)	Phase I, recruiting
	NCT02130466	Pembrolizumab	Advanced Melanoma	Monotherapy Combination: • Trametinib • Dabrafenib • Trametinib and Dabrafenib	Phase I / II, active, not recruiting
	NCT02494583	Pembrolizumab	Advanced gastric or gastroesophageal junction adenocarcinoma	Monotherapy Combination: • 5-FU • Cisplatin	Phase III, active, not recruiting
PD-L1	NCT01633970	Atezolizumab	Locally Advanced or Metastatic Solid Tumors	Combination: • Bevacizumab • Bevacizumab + 5-FU • Carboplatin + Paclitaxel • Carboplatin + Pemetrexed • Carboplatin + Nab-paclitaxel • Nab-paclitaxel	Phase I, active, not recruiting

**Table 3:** Anti-LAG-3, anti-TIM-3, anti-PD-L1, -L2, -VISTA and anti-TIGIT immune checkpoint blockade tested in clinical trials as mono- and/or combination therapies.

**Target**	**Study ID number**	**Drug name**	**Indication(s) (cancer type/subtype)**	**Setting**	**Stage of development and status**
LAG-3	NCT02061761	BMS-986016	Hematologic malignancies	Monotherapy Combination: • Nivolumab (BMS-936558)	Phase I / II, recruiting
	NCT02966548	BMS-986016	Advanced solid tumors	Monotherapy Combination: • Nivolumab (BMS-936558)	Phase I, recruiting
	NCT03005782	REGN3767	Advances cancers	Monotherapy Combination: • REGN2810 (anti-PD1)	Phase I, recruiting
	NCT01968109	BMS-986016	Solid tumors	Monotherapy Combination: • Nivolumab (BMS-936558)	Phase I / II, recruiting
TIM-3	NCT02817633	TSR-022	Advanced solid tumors	Monotherapy Combination: • anti-PD-1	Phase I, recruiting
	NCT02608268	MBG453	Advanced malignancies	Combination: PDR001 (anti-PD-1)	Phase I / II, recruiting
	NCT03066648	MBG453	Hematologic malignancies	Monotherapy Combination: • PDR001 (anti-PD-1) • and/or PDR001 combined with decitabine	Phase I, recruiting
PD-L1, PD-L2, VISTA	NCT02812875	CA-170	Advanced solid tumors and lymphomas	Monotherapy	Phase I, active, not recruiting
TIGIT	NCT03119428	OMP-313M32	Locally advanced or metastatic solid tumors	Monotherapy Combination: • Nivolumab	Phase Ia/b, active, not recruiting
	NCT02913313	BMS-986207	Advanced or metastatic solid cancers	Monotherapy Combination: • Nivolumab	Phase I/II, recruiting

## Conclusion

In contrast to chemotherapy or oncogene-targeted therapies, cancer immunotherapy relies on promoting an anti-cancer immune response, a dynamic process involving several mechanisms and cross-talks between different cellular compartments. When neutralizing immune factors, one of the major issues is the off-target effects, which lead to unforeseen complications for patients. Many soluble factors are involved in several signaling pathways, thus the targeting of one specific soluble factor may disturb signaling pathways that were not initially meant to be targeted. Moreover, they are not unique to the tumor and TME, but mandatory for maintaining homeostasis. Following a series of breakthroughs, we are witnessing an acceleration of the research in the field of cancer immunotherapy, which can simultaneously target several TME abnormalities in the clinical setting. For instance, optimal cancer therapy with inhibition of the PD-1/PD-L1 axis should include: (1) modulation at the tumor site due to the localized expression pattern of PD-L1 in the TME, (2) targeting of elevated immune inhibitory cytokines (IL10, TGF-β), tumor metabolites and regulatory cells, and (3) rescue of the tolerated tumor immunity (177). Since 2011, the FDA has approved several agents as a standard of care therapy in oncology and hematology (Supplementary Table 1). Active research focuses on the integration of combinations that may boost the response rate to immunotherapy, which still remains low. An unmet need is the identification of predictive biomarkers for immunotherapy that could optimize patients' stratification and administration of combination therapies. This can be done through the discovery of “biomarkers of response” in patients that showed clinical benefit. Predictive factors for immunotherapy are being actively investigated for establishing an “immune signature” of tumors that defines genetic, molecular and functional profiles of immune cells present in the TME (178). The patient-specific landscape of the TME can be appreciated by using “immunograms” as integrated biomarkers, obtained by capturing the immune profile with next-generation sequencing data. The development of personalized biomarker profiles, and thus, the characterization of microenvironmental features and their changes during treatment, represents a comprehensive knowledge which will become a valuable resource for optimal personalized immunotherapy and patient monitoring (179). In the era of precision medicine, tailoring cancer immune-interventions combined with other comprehensive approaches may pave the way toward an appropriate modulation of anti-cancer immune responses, on the basis of the genetic and immunological analyses in each patient. Combination approaches altering the TME and/or decreasing immunosuppression along with strategies that are countervailing insufficient tumor immunogenicity and antigenicity may be required to achieve effective tumor control.

## Author Contributions

BG and EA designed the manuscript. BG wrote the manuscript with inputs from CM and EA. CC, CM, SR, EA, and BG reviewed and approved the manuscript.

### Conflict of Interest

EA is co-founder and CEO of Veana Therapeutics, Inc. The remaining authors declare that the research was conducted in the absence of any commercial or financial relationships that could be construed as a potential conflict of interest.

## Abbreviations

ICB: Immune checkpoint blockade
TME: Tumor microenvironment
Tregs: regulatory T cells
CTL: cytotoxic T lymphocytes IFN-γ: interferon-γ
IDO: indoleamine 2,3- dioxygenase
CCL: C-C Motif Chemokine Ligands
TGF- β: Transforming growth factor- β
NK: natural killer
DC: dendritic cells
NSCLC: non-small-cell lung carcinoma
MDSCs: myeloid-derived suppressor cells
mAb: monoclonal antibodies
OS: overall survival
IL: Interleukin
APC: antigen presenting cells
TLR: Toll-like receptor
MHC-II: major histocompatibility complex II
TAMs: tumor-associated macrophages
VEGF: Vascular endothelial growth factor
FDA: Food and Drug Administration
PFS: progression free survival
NSCLC: Non-small-cell lung carcinoma
M-CSF: macrophage colony-stimulating factor
CAF: cancer-associated fibroblasts
ICIs: immune checkpoint inhibitors
TNF-α: tumor necrosis factor
iNOS: inducible nitric oxide synthase
PDE-5: Phosphodiesterase type 5 inhibitors
cAMP: cyclic adenosine monophosphate
cGMP: cyclic guanosine monophosphate
HNSCC: Head and neck squamous cell carcinomaSHP2: Src homology 2 domain-containing tyrosine phosphatase 2
PD-1: Programmed cell death protein 1
PD-L1: programmed death-ligand 1
RCC: renal cell carcinoma
LXR: liver X receptor
TIM-3: T cell immunoglobulin and mucin 3
LAG-3: Lymphocyte-activated gene-3
ITIM: immunoreceptor tyrosine-based inhibition motif
VISTA: V-domain Ig-containing suppressor of T cell activation or PD-1 homologue
TIGIT: T cell immunoglobulin and immunoreceptor tyrosine-based inhibitory domain.
