# Supplementary material for: Reverting Immune Suppression to Enhance Cancer Immunotherapy
Source: Front Oncol. 2020 Jan 21;9:1554. doi: 10.3389/fonc.2019.01554 (PMC6985581; doi:10.3389/fonc.2019.01554)
Supplement: Supplementary file 1 [file Table_1.pdf]

Supplementary Material

1 Supplementary Table 1

Timeline of some immunotherapeutic agents approved as a standard of care therapy in oncology and hematology

Drug / Inhibitor	Indication (s) (cancer type / subtype)	FDA Approvals	
**Ipilimumab (anti-CTLA-4, Yervoy, Bristol-Myers Squibb Company)**	Treatment of advanced melanoma	**March**	**2011**
**Pembrolizumab (anti-PD-1, KEYTRUDA, MK-3475, Merck & Co., Inc.)**	Treatment of advanced or unresectable melanoma patients who are no longer responding to other therapy	**September**	**2014**
**Nivolumab (anti-PD-1, OPDIVO, Bristol-Myers Squibb Company)**	Treatment of metastatic melanoma patients who are no longer respond to other drugs	**December**	**2014**
**Nivolumab**	Treatment of metastatic squamous non-small cell lung cancer (NSCLC) with progression on or after platinum-based chemotherapy.	**March**	**2015**
**Nivolumab**	Treatment of metastatic non-small cell lung cancer patients whose disease progressed during or after platinum-based chemotherapy.	**October**	**2015**
**Pembrolizumab**	Treatment of non-small cell lung cancer patients whose tumors express PD-L1 and who have failed treatment with chemotherapeutic agents	**October**	**2015**
**Nivolumab**	Treatment of metastatic renal cell carcinoma patients who have received a certain type of prior therapy	**November**	**2015**
**Atezolizumab (anti-PD-L1, TECENTRIQ, Genentech Oncology)**	Treatment of locally advanced or metastatic urothelial carcinoma that has progressed after platinum-containing chemotherapy	**May**	**2016**
**Atezolizumab (anti-PD-L1, TECENTRIQ, Genentech Oncology)**	Treatment of metastatic NSCLC whose disease progressed during or following platinum-containing chemotherapy	**October**	**2016**
**Nivolumab**	Treatment of locally metastatic urothelial carcinoma patients who have disease progression during or following platinum-containing chemotherapy or neoadjuvant or adjuvant treatment with a platinum-containing chemotherapy.	**February**	**2017**
**Avelumab (BAVENCIO, EMD Serono, Inc.)**	Treatment of metastatic Merkel-cell carcinoma patients	**March**	**2017**
**Pembrolizumab**	Treatment of patients with refractory classical Hodgkin lymphoma, or those who have relapsed after three or more prior lines of therapy.	**March**	**2017**
**Atezolizumab**	Treatment of advanced bladder cancer patients who are unable to receive initial treatment with cisplatin chemotherapy	**April**	**2017**
**Avelumab**	Treatment of locally advanced or metastatic urothelial carcinoma in first line setting	**May**	**2017**
**Avelumab**	Treatment of locally advanced or metastatic urothelial carcinoma whose disease progressed during or following platinum-containing chemotherapy or within 12 months of neoadjuvant or adjuvant platinum-containing chemotherapy.	**May**	**2017**
**Pembrolizumab**	Treatment of patients with unresectable or metastatic solid tumors harboring a specific genetic feature	**May**	**2017**
**Nivolumab**	Treatment of hepatocellular carcinoma patients after sorafenib therapy	**September**	**2017**
**Nivolumab**	Combined with ipilimumab for the treatment of intermediate or poor risk, previously untreated advanced renal cell carcinoma	**April**	**2018**
**Pembrolizumab**	Treatment of:<br>- Recurrent or metastatic cervical cancer with disease progression on or after chemotherapy whose tumors express PD-L1<br>- Refractory primary mediastinal large B-cell lymphoma	**June**	**2018**
**Nivolumab**	Treatment of metastatic small cell lung cancer patients whose cancer has progressed after platinum-based chemotherapy and at least one other line of therapy	**August**	**2018**
**Pembrolizumab**	First-line treatment of patients with metastatic, non-squamous NSCLC with no EGFR or ALK genomic tumor aberrations in combination with chemotherapy	**August**	**2018**
